# Chronic myelogenous leukemia coexpressing V-e16a2, V-e13a2, e13a2, and e14a2 *BCR::ABL1* fusion transcripts: a case report and review of the literature

**DOI:** 10.3389/fonc.2024.1518387

**Published:** 2024-12-12

**Authors:** Yang Xu, Yuying Fan, Sijin Liu, Jianmei Chang, Caifeng Guo, Lanhui Chen, Wenzheng Guo, Jinwen Dang, Hongwei Wang, Yanhong Tan

**Affiliations:** Institute of Hematology, The Second Hospital of Shanxi Medical University, Taiyuan, China

**Keywords:** chronic myelogenous leukemia, BCR::ABL1, e16a2 transcripts, variant transcript, tyrosine kinase inhibitors

## Abstract

The coexistence of three or more transcripts in one patient with chronic myeloid leukemia (CML) is rarely reported. Thus, the disease progression and drug response are still unknown. This case report aimed to explore the drug response of CML with variant transcripts and to enrich the clinical treatment of rare types of CML. A 66-year-old Chinese female patient was diagnosed with chronic myeloid leukemia-chronic phase (CML-CP) expressing four *BCR::ABL1* transcripts, including variant e16a2(V-e16a2), variant e13a2(V-e13a2), classical e13a2, and e14a2 transcripts. The patient was treated with flumatinib, a tyrosine kinase inhibitor (TKI).The variant transcripts reported exhibited a favorable response to TKI, and attention should be directed toward monitoring variant transcripts.

## 1 Introduction

The presence of the Philadelphia (Ph) chromosome, which is due to the reciprocal translocation t(9;22) (q34;q11), is a distinctive characteristic of chronic myelogenous leukemia (CML) (1–3). This translocation leads to a breakpoint cluster region (BCR)-Abelson 1 (ABL1) fusion transcript. In general, as the breakpoint region occurs in M-bcr, m-bcr, and μ-bcr, fusion transcripts e13a2 (b2a2), e14a2 (b3a2), e1a2, and e19a2 are generated, respectively (4, 5). Tyrosine kinase inhibitors (TKIs) were strategically crafted to selectively target the fusion protein, aiming to impede its enzymatic activity. This targeted inhibition has resulted in a noteworthy frequency of remission and improved the survival rates among patients diagnosed with CML (6).

However, evidences indicate instances characterized by an atypical clinical presentation, predominantly associated with alternative transcripts. These occurrences are observed in 1% of patients with CML, and their clinical significance is under investigation (7–10). Such infrequent variant transcripts can induce phenotypic variability and impact the response to TKI therapy (11). The current literature reported that abnormal splices involved are BCR exons 1, 6, 8, 12, 13, 14, and 19, as well as ABL exons 2 and 3 (7, 12–17). Herein, we describe a rare case of CML involving abnormal splicing of BCR exons 16 and 13 and ABL exon 2. This splicing event resulted in the coexpression of four *BCR::ABL1* transcripts of variants e16a2 and e13a2 and classical e13a2 and e14a2. This patient was followed up for 14 months to observe her response to TKI after treatment with flumatinib.

## 2 Case report/case presentation

A 66-year-old female was admitted to the Hematology Department in November 2021 because of dizziness and fatigue. Laboratory examinations revealed the hemoglobin (Hgb) level of 36 g/L, white blood cell count of 74.23 × 10^9^/L, and platelet count of 1,437 × 10^9^/L. Bone marrow imaging showed extremely active proliferation, with a markedly increased granulocyte lineage, predominantly in the middle and late juvenile granulocytes, and an increased number of megakaryocytes, with small cytosols and few lobes. Immunophenotyping results revealed that lymphocytes, monocytes, granulocytes, and primitive cells accounted for 8.3%, 1%, 70.9%, and 3.4%, respectively. The proportion of primitive cells and granulocytes increased, with positive expression of CD34, CD117, CD56, CD38, and CD13. Fluorescence *in situ* hybridization analysis using dual-fusion probes showed the *BCR::ABL1* classical fusion signal in 99% of 500 cells counted, and cytogenetic analysis was positive for 46, XX, t(9;22)(q34;q11). Thus, the patient was diagnosed with chronic phase CML. The Sokal and ELTS scores indicated high risk.

**Figure 1:**
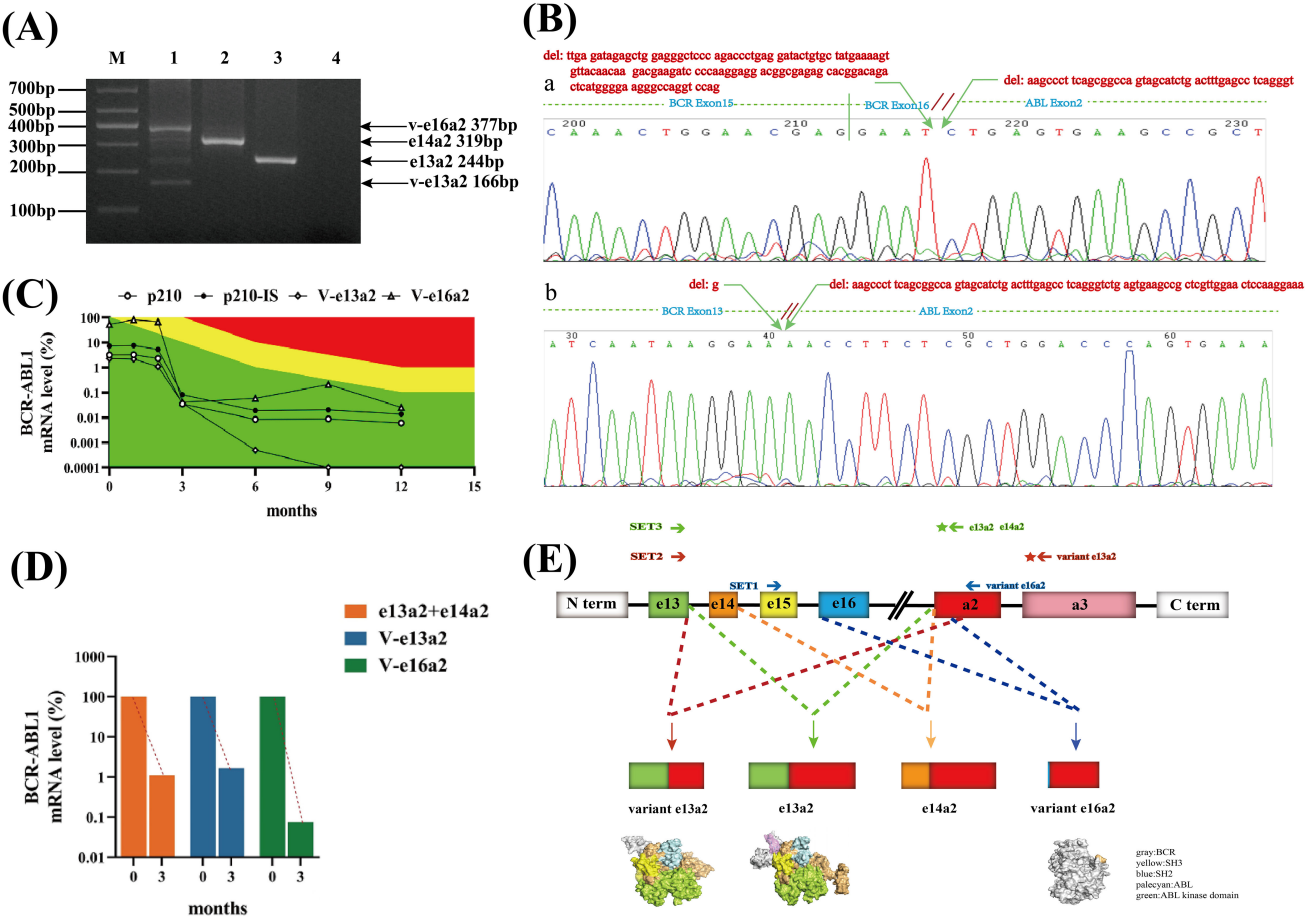
Identification and screening of *BCR::ABL1* coexpressing variant e16a2(V-e16a2), variant e13a2(V-e13a2), e13a2, and e14a2 transcripts and structure diagram. **(A)** Electrophoretogram of different *BCR::ABL1* transcripts in a patient with CML. M: marker, Lane 1: coexpressing variant e16a2(V-e16a2)(377bp), variant e13a2(V-e13a2)(166bp), e13a2(244bp), and e14a2(319 bp), Lane 2: e14a2(319bp), Lane 3: e13a2(244bp), Lane 4: blank. **(B)** Sequencing results of various transcripts. a: variant e16a2(V-e16a2), b: variant e13a2(V-e13a2). **(C)** Dynamic monitoring results of the expression of four transcripts of the *BCR::ABL1* fusion gene in a CML patient. Green area: optimal response, yellow area: nonoptimal response, red area: resistance. **(D)** Diagram of changes in early molecular reaction rates. **(E)** Schematic of the generation and protein structure prediction of various *BCR::ABL1* transcripts.

Furthermore, nest-RT-PCR was used to detect the *BCR::ABL1* fusion gene. This method can detect most transcripts, as described in the literature (18). Surprisingly, we not only detected classical e13a2 (244bp) and e14a2 (319bp) transcripts but also found abnormal bands of 377 and 166 bp in size, respectively, in the same lane (**Figure 1A**). Abnormal-sized bands were excised and subjected to Sanger sequencing. The sequencing results (**Figure 1B**) revealed a fusion of a 128-bp deletion at the C-terminal end of exon 16 of the BCR gene with a 44-bp deletion at the N-terminal end of exon 2 of the ABL1 gene, forming an atypical e16a2 transcript. Because of the frameshift mutation, the transcript encodes a 964-amino-acid truncated protein and almost loses most of the ABL region, including the kinase domain. Additionally, a 1-bp deletion at the C-terminal end of exon 13 of the BCR gene was fused with a 77-bp deletion at the N-terminal end of exon 2 of the ABL1 gene, resulting in the deletion of 26 amino acids in the frame and the formation of an atypical e13a2 transcript. Fortunately, the integrity of the ABL kinase domain remained uncompromised.

The patient was diagnosed and started on flumatinib 600 mg QD for treatment. To understand how well these abnormal transcripts responded to TKI, their cytogenetic and molecular biology monitoring results were regularly followed up. Because the detection range of conventional quantitative PCR could not cover abnormal transcript, special primers and probes were designed to detect each transcript. The results of typical transcripts were expressed on the International Scale (IS) whereas it was not possible to report the results of atypical transcripts on the IS. The SET1 primer pair (BCR gene exon15F:5′ GGATTCCTTTGGGTATTTTGTGA 3′ and ABLexon2R1:5′ TGGGGTCATTTTCACTGGGT 3′) monitored the V-e16a2 transcript, while the SET2 primer pair (BCR exon13F: 5′-CCGCTGACCATCAATAAGGAA-3′, ABLexon3R: 5’-TCACACCATTCCCCATTGTG-3’ and probeA3: 5′-CCGGGTCTTAGGCTATA-3′) monitored the V-e13a2 transcript. Classical primer set3 (BCR exon13F: 5′-CCGCTGACCATCAATAAGGAA-3′, ABLexon2R2:5′-CACTCAGACCCTGAGGCTCAA -3′, and probeA2:5′-CCCTTCAGCGGCCAGTAGCATCTGA-3′) was used to amplify P210 (total e13a2 and e14a2) (**Figure 1E**). The patient was followed up for 14 months, and the expression of each transcript (shown in **Figure 1C**) demonstrated that all four transcripts of the patients showed a decreasing trend with the prolongation of the medication time, presenting a good response to flumatinib. The V-e13a2 transcript was undetectable by RT-qPCR after 9 months. However, the rate of decline indicates that the V-e16a2 transcript may be more sensitive than the V-e13a2 transcript at the early stage (**Figure 1D**). The total expression of e13a2 and e14a2 was not high at baseline, and *BCR::ABL1*/ABL-IS was always optimal after treatment.

## 3 Discussion

The *BCR::ABL1* fusion gene generated by chromosome t(9;22) translocation is an important mechanism in the development of Ph+ leukemias, and *BCR::ABL1* exhibits diversity at the mRNA and protein levels due to different breakpoints and the involvement of alternative splicing. With the widespread use of various sequencing technologies, rare *BCR::ABL1* transcripts other than classical transcripts have been discovered and confirmed. These variant transcripts are generally believed to exist because of the following two main ways. One mechanism involves the rearrangement of atypical breakpoint regions of the BCR and ABL genes resulting in the formation of fusion transcripts such as e8a2, e15a2, e6a2, and e1a3. Another reason is alternative or abnormal splicing at the mRNA level, whose variant transcripts are mostly caused by deletions or insertions of partial or full sequences in the exons or intros of the BCR and ABL1 genes. Most of these atypical transcripts reported so far are accompanied by typical transcripts (19), which support this formation mechanism. The patient first reported here harbored two atypical transcripts due to partial exon deletions in the BCR and ABL1 genes. It was hypothesized that the breakpoints were located in BCR intron 16 and ABL intron 1. Multiple abnormal transcripts subsequently emerged as a result of abnormal splicing events at the mRNA level (**Figure 1E**), with coexpression of the normal transcripts. Furthermore, a fusion of BCR e16 with ABL a2 was also identified for the first time.

The clinical significance and prognostic values of atypical transcripts in ph-positive leukemias are still unclear from the current studies, and most of them are individual case reports that lack reproducibility and are highly heterogeneous. Abnormal splicing of genes often alters their functions (20). Therefore, whether the proteins encoded by these atypical transcripts are oncogenic, with or without kinase activity, and how they respond to TKIs are of special interest for individualized patient treatment. Patients carrying the atypical *BCR::ABL1* e6a2 fusion transcript are depicted as having an aggressive clinical course and are associated with a poor prognosis (21). Manzella et al. (21) described a case of a female patient with CML carrying the *BCR::ABL1* e6a2 fusion transcript. Treatment with nilotinib, a second-generation TKI, was effective in this patient. In other cases with rare transcripts, the impact on outcome remains unclear because of their rarity and lack of data. This indicates that the differential response of abnormal transcripts to TKIs may depend on alterations in their structural or functional domains. Furthermore, the form of the transcript should be considered when deciding on the optimal treatment approach. In this report, the patient’s response to TKI was closely monitored, which may provide a basis for the treatment of more specific cases. After treating the patient with flumatinib 600 mg/day, both atypical e16a2 and e13a2 transcripts and classical e14a2 and e13a2 transcripts showed a sensitive response. The v-e16a2 transcript with a higher proportion of initial expression volume had a faster decline rate at the early stage (**Figure 1D**). In terms of protein structure (**Figure 1E**), compared with the classical e13a2 transcript, the v-e13a2 transcript was analyzed with an in-frame deletion mutation that preserved the intact ABL1 functional domain, and the ATP-binding site of the ABL1 kinase was not disrupted. The 26 missing amino acids primarily encode a critical N-terminal cap segment of the ABL gene (pink part), which helps keep the SH3-SH2 substructure in a self-inhibitory state and locks it to the distal surface of the kinase domain. Deletion of the mutant may prolong the activity of ABL kinase by preventing it from returning to a self-inhibited state (22). This could lead to the possibility that v-e13a2 may exhibit heightened kinase activity. In terms of drug response, the response of this transcript to TKIs may be as effective as that in classical transcripts. In this patient, the truncated e16a2 transcript resulted in the termination of the ABL region after encoding only two amino acids, leading to a loss of almost all ABL functional domains; however, its presence was consistently detected during dynamic monitoring of the patient and gradually decreased over time with TKI. Although the v-e16a2 transcript does not directly interact with TKIs, its reduced expression likely stems from the reduction in alternative splicing events presented by the decreased *BCR::ABL1*-positive cells in patients treated with TKI. This hypothesis gains support from the consistent trend observed in other transcripts.

Molecular monitoring of *BCR::ABL1* levels by RT-qPCR has been standardized and the standardized system is widely used in different laboratories (23). Atypical transcripts are not efficiently detected by RT-qPCR and molecular results cannot be expressed on IS as for common transcripts. In these cases, patient-specific primers and probes are needed to perform a dynamic RT-qPCR monitoring. Moreover, conventional multiplex RT-PCR usually fails to detect uncommon *BCR::ABL1* rearrangements because of the generation of atypical PCR products, which are often interpreted as nonspecific and may lead to misdiagnosis, thus excluding the patient from targeted therapy. In conclusion, qualitative RT-PCR remains an indispensable test and attention should be paid to the interpretation of the results. Besides classical techniques, high sensitivity technologies, such as digital PCR, can be useful to monitor atypical transcripts and to achieve the goal of truly individualized treatment for CML patients.

## Data Availability

The original contributions presented in the study are included in the article/supplementary material. Further inquiries can be directed to the corresponding authors.
